# Association between HIV Serostatus and premalignant cervical lesions among women attending a cervical cancer screening clinic at a tertiary care facility in southwestern Uganda: a comparative cross-sectional study

**DOI:** 10.1186/s12905-024-03108-w

**Published:** 2024-04-27

**Authors:** Justus Kirabira, Musa Kayondo, Stephen Mayanja Bawakanya, Edirisa Juniour Nsubuga, Fajardo Yarine, Alexcer Namuli, Rita Namugumya, Christine Hilda Natulinda, Raymond Atwine, Abraham Birungi, Henry Mark Lugobe, Leevan Tibaijuka, Dean Kisombo, Mark Jjuuko, David Collins Agaba, Pascal Saturday, Subira Mlangwa Atupele, Matthew Tumusiime, Richard Migisha, Rogers Kajabwangu

**Affiliations:** 1 Department of Obstetrics and Gynecology, Mbarara University of Science and Technology, Mbarara City, Uganda; 2 Department of Obstetrics and Gynecology, Mbarara Regional Referral Hospital, Mbarara City, Uganda; 3 Ministry of Health, Kampala, Uganda; 4 Department of Physiology, Mbarara University of Science & Technology, Mbarara City, Uganda; 5 Department of Pathology, Mbarara University of Science & Technology, Mbarara City, Uganda

**Keywords:** HIV, High-grade squamous intraepithelial lesion, Low-grade squamous intraepithelial lesion, Papanicolaou smears, Uganda

## Abstract

**Background:**

Uganda has approximately 1.2 million people aged 15–64 years living with human immunodeficiency virus (HIV). Previous studies have shown a higher prevalence of premalignant cervical lesions among HIV-positive women than among HIV-negative women. Additionally, HIV-infected women are more likely to have human papilloma virus (HPV) infection progress to cancer than women not infected with HIV. We determined the prevalence of premalignant cervical lesions and their association with HIV infection among women attending a cervical cancer screening clinic at Mbarara Regional Referral Hospital (MRRH) in southwestern Uganda.

**Methods:**

We conducted a comparative cross-sectional study of 210 women aged 22–65 years living with HIV and 210 women not living with HIV who were systematically enrolled from March 2022 to May 2022. Participants were subjected to a structured interviewer-administered questionnaire to obtain their demographic and clinical data. Additionally, Papanicolaou smears were obtained for microscopy to observe premalignant cervical lesions. Multivariate logistic regression was performed to determine the association between HIV status and premalignant cervical lesions.

**Results:**

The overall prevalence of premalignant cervical lesions in the study population was 17% (*n* = 72; 95% C.I: 14.1–21.4), with 23% (*n* = 47; 95% C.I: 17.8–29.5) in women living with HIV and 12% (*n* = 25; 95% C.I: 8.2–17.1) in women not living with HIV (*p* < 0.003). The most common premalignant cervical lesions identified were low-grade squamous intraepithelial lesions (LSIL) in both women living with HIV (74.5%; *n* = 35) and women not living with HIV (80%; *n* = 20). HIV infection was significantly associated with premalignant lesions (aOR: 2.37, 95% CI: 1.27–4.42; *p* = 0.007).

**Conclusion:**

Premalignant cervical lesions, particularly LSILs, were more common in HIV-positive women than in HIV-negative women, highlighting the need to strengthen the integration of cervical cancer prevention strategies into HIV care programs.

## Introduction

Cervical cancer is the fourth most common cancer among women worldwide [1, 2] and the leading cause of cancer-related deaths in developing countries [3–5]. It is preventable through vaccination, screening and treatment for human papilloma virus (HPV) infection and premalignant cervical lesions [5]. Approximately 570,000 cases of cervical cancer and 311 000 deaths from the disease occurred worldwide in 2018 [6]. The incidence of cervical cancer in Uganda is one of the highest in the world, with an age-standardized rate of 54.8 per 100,000 women per year [7], and the prevalence of premalignant cervical lesions is 22% [8].

A premalignant cervical lesion is the presence of proliferative dysplastic cells confined to the epithelium of the cervix [9]. These lesions may be classified based on the Bethesda classification, which includes atypical cells, low-grade squamous intraepithelial lesions (LSILs) and high-grade squamous intraepithelial lesions (HSILs) [10, 11]. Studies have shown that 10% of patients who have LSIL progress to HSIL [12]. Other classifications include cervical intraepithelial neoplasia (CIN), which can be further classified as CIN 1, 2, or 3 according to the level of dysplasia, where CIN 1 is a low-grade squamous intraepithelial lesion, while CIN 2 or 3 are high-grade squamous intraepithelial lesions [13]. Approximately 1–2% of women worldwide are estimated to develop CIN 2 and 3 each year, and the prevalence is reportedly high in human immunodeficiency virus (HIV)-positive women, at 10% [14]. These premalignant cervical lesions are preceded by persistent infection with high-risk human papilloma virus (HPV), especially types 16 and 18 [15]. This leads to the integration of HPV into host deoxyribonucleic acid (DNA), which leads to resistance to apoptosis, causing uncensored cell growth and hence progression to premalignant cervical lesions [16]. Premalignant cervical lesions are easier to manage before progression to cervical cancer occurs [17, 18].

Premalignant cervical lesions progress in stages from low-grade squamous intraepithelial lesions to high-grade squamous intraepithelial lesions and eventually to cancer [9]. The overall progression to cancer takes 10–20 years [9]. HIV-positive women have higher rates of progression to carcinoma and lower rates of regression than HIV-negative women. Compared with HIV-negative women, HIV-positive women have an increased risk of HPV infection and precancerous lesions [19]. HIV-positive women are approximately 3 times more likely to have their HPV infection progress to HSIL than HIV-negative women are [19]. This is because of a decrease in T-cell surveillance in HIV infection, which allows HPV replication, resulting in persistence of HPV infection, the accumulation of mutations in infected cells, and the subsequent proliferation of dysplastic cells [20]. HPV infection among HIV-positive women is strongly associated with low cluster of differentiation 4 (CD4) counts and high HIV viral load [21]. Women infected with HIV have a higher risk of incidence and persistence of human papillomavirus (HPV)-associated premalignant cervical lesions than women without HIV because HIV increases the entry of HPV into host DNA and enhances cell dysplasia [17, 18].

HIV-associated premalignant cervical lesions are thought to progress through the microsatellite instability pathway, whereas HIV-negative lesions progress through loss of heterozygosity. Interactions are likely via viral proteins, with HIV proteins enhancing the effectiveness of HPV proteins and possibly contributing to cell cycle disruption. Dysregulation of the cellular and humoral arms of the local and systemic immune systems may ensure disease progression [22]. The HIV provirus encodes structural retrovirus proteins that include gag, pol, env, tat, rev, vif, nef, vpr, and vpu. With regard to premalignant cervical lesions, tat and rev are two control mechanisms for viral gene expression. The tat protein enhances the transcription of viral genes, whereas rev acts post-transcriptionally, shuttling viral messenger ribonucleic acid (mRNA) from the nucleus to the cytoplasm [23].

In HIV-negative women with competent immune systems, most infections are cleared spontaneously because of a cell-mediated immune response regulated by CD4 + lymphocytes. However, in HIV-infected individuals, there is a higher risk of persistent HPV infection largely due to their impaired ability to clear HPV infection; hence, they are at increased risk of developing cervical dysplasia [16]. HIV-positive women with low CD4 lymphocyte counts have the highest prevalence of HPV infection and have shown higher detection rates of mixed HPV types with persistent infection [18]. Increased access to antiretroviral therapy (ART) has increased the life expectancy of women living with HIV, but many of these women remain susceptible to high-risk HPV infection, persistent disease, cervical lesion incidence and progression to carcinoma of the cervix [24]. The association between HIV and premalignant cervical lesions is moderated by several other factors, such as early age at first sexual intercourse, multiple sexual partners, previous history of sexually transmitted infections (e.g., chlamydia infections), level of education, oral contraceptive use, multiparity, smoking and low body mass index [25–28].

The prevalence of HIV in women living in Uganda is 7.2%, and Mbarara city has a prevalence of 7.9%, which is the second highest prevalence among districts in Uganda [29]. In Mbarara City, during July–September 2020, 18 (75%) of the women who had cervical cancer were HIV positive, and 10 (42%) of those women were referred from the cervical cancer screening clinic at Mbarara Regional Referral Hospital (MRRH) [30]. However, in our setting, limited studies have been conducted to understand the association of premalignant cervical lesion among HIV positive women and HIV negative women. Such information is crucial for better informing logistical planning, early identification of lesions to avoid progression to cancer, and addressing the other modifiable risk factors that are associated with premalignant cervical lesions. Thus, this study investigated the prevalence of premalignant cervical lesions and their association with HIV status among women attending the cervical cancer screening clinic at the MRRH.

## Methods

### Study design and period

This was a comparative cross-sectional study conducted among women attending the cervical cancer screening clinic of MRRH over a period of three months from March 2022 to May 2022.

### Study area

The study was conducted at the cervical cancer screening clinic of MRRH, which is a government tertiary hospital that also serves as the teaching hospital for Mbarara University of Science and Technology (MUST). The hospital is located in Mbarara City, southwestern part of Uganda, approximately 260 km southwest of Kampala, the capital city of Uganda. It provides preventive, diagnostic, curative and rehabilitation services to patients from the 13 catchment districts, which have a population of approximately 5 million people in southwestern Uganda.

The cervical cancer screening runs daily, with an average daily attendance of 15 patients equally distributed among HIV-positive and HIV-negative women aged 22 to 65 years. The clinic is run by specialist physicians and midwives, and it offers cervical cancer screening using visual inspection with *acetic acid* or *Lugol’s* iodine and Papanicolaou (Pap) smears, especially for postmenopausal women. Ablative therapy is offered for precancerous lesions by thermocoagulation and loop electrosurgical procedures.

Colposcopy-guided biopsy, endocervical curettage and diagnostic conization are also performed [31]. Once the Pap smears are collected, they are sent to the histopathology department of the hospital for staining and reading by a pathologist at Mbarara Regional Referral hospital and every 10th smear examined by an independent pathologist at Mulago Specialized Women and Neonatal Hospital.

### Study population, sample size and sampling

The study population included women who attended the cervical cancer screening clinic at MRRH. Women who were in their menses, who had active vaginal bleeding and who had suspicious lesions for cervical cancer were excluded. A sample size of 420 (210 HIV-positive and 210 HIV-negative women) was estimated using the formula for comparison of two proportions [32], with a 95% confidence interval (CI), a power of 80%, a prevalence of premalignant cervical lesions of 12.6% in HIV-positive women and 4.6% among HIV-negative women [33], and a 10% non-satisfactory Pap smear. On average, an estimated 900 women are screened for cervical cancer at MRRH every three months [30]. Dividing 900 by the sample size (420) gives a sampling interval (*k*) of two. Considering the two strata of HIV-positive and HIV-negative women, we used systematic sampling to recruit every second woman in each stratum following their order of coming to the clinic until the sample size was reached in each group. The rotary method of simple random sampling was used to select the first participant in each stratum. If the selected participant was not eligible for the study, we chose the next immediate woman whom we subjected to the sampling interval to obtain the next participant. This was performed until a maximum sample size of 420 (210 from each group) was obtained.

### Inclusion and exclusion criteria

All women who came for cervical cancer screening at the cervical cancer screening clinic at Mbarara Regional Referral Hospital were potential participants but excluded if they had menses or had active vaginal bleeding, or visible suspicious lesions for cervical cancer. This is because the red blood cells contained in menses and vaginal bleeding interfere with reading the slides under a microscope where as those with suspicious lesions, biopsy was recommended instead.

### Variables

#### Dependent variable

The dependent variable was the presence of a premalignant cervical lesion on Pap smear examination based on the Bethesda 2014 classification system [10, 11, 34]. Briefly, lesions were considered premalignant if they had atypical cells, low-grade squamous intraepithelial lesions (LSILs), high-grade squamous intraepithelial lesions (HSILs), or atypical glandular cells.

#### Independent variables

We collected data on medical factors, which included HIV status, body mass index (BMI), and history of other sexually transmitted infections (STIs). The socio-demographic factors assessed were age, religion, education level, residence, employment status, marital status and smoking status (ever smoked or not). In addition, we obtained data on reproductive health factors, including parity, age at first sexual intercourse, abortion history, number of sexual partners in the past 20 years and oral contraceptive use.

### Data collection

**Fig. 1:**
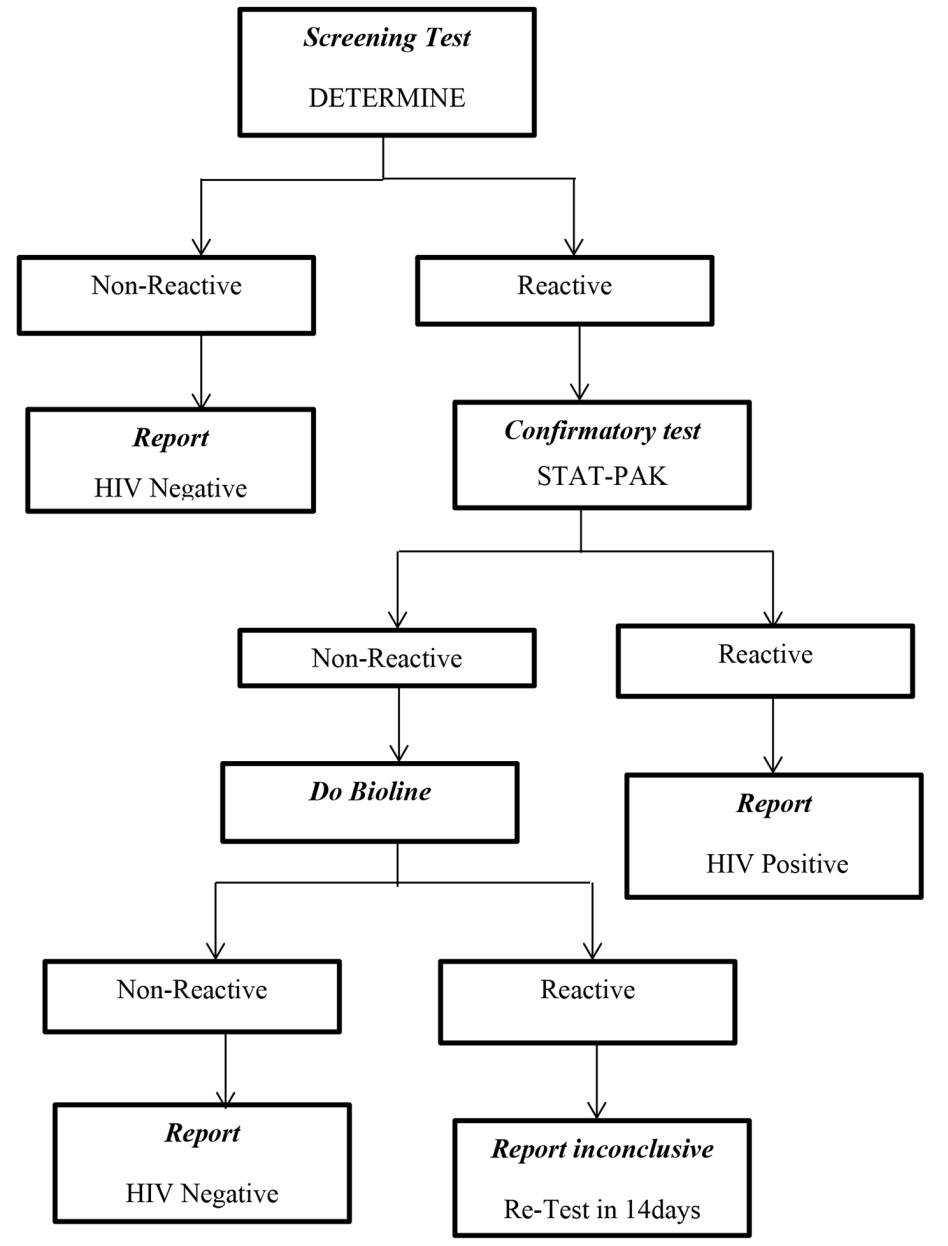
Serial HIV testing algorithm for individuals older than 18 months in Uganda

After obtaining written informed consent, socio-demographic data was collected using a questionnaire. Information on HIV serostatus was obtained by looking at documented evidence of participants HIV status or testing using the national HIV algorithm as shown in Fig. 1 [35]. These women were then grouped into two different strata depending on their HIV status (HIV-positive and HIV-negative). Research assistants then interviewed the respondents face to face using a pretested structured coded questionnaire, after which the respondents were then taken for Pap smear collection.

Weight and height measurements were performed using standardized scales and stadiometers according to the hospital standard operating procedures.

All anthropometric measurements were taken twice, and the mean was entered into the questionnaire. The data collection was supervised, and the completed questionnaires were reviewed for completeness on a daily basis.

#### Pap smear collection

A pap smear was collected as per standard operating procedures by a qualified study staff, stained and read by a histopathologist. Slides were read by two readers, any discrepancy in results was read by a third reader who acted as a tie breaker.

#### Interpretation of pap smear results

We used the Bethesda 2014 classification system for interpretation of precancerous cervical smears stained by the Pap method [10–12].

### Quality control

Pap smears were read by a pathologist at Mbarara Regional Referral hospital and every 10th smear was examined by an independent pathologist at Mulago Specialized Women and Neonatal Hospital. Inter rater reliability testing was done by calculating Cohen’s kappa coefficient and results were found consistent. All participants were screened for HIV status.

### Data analysis

The data were collected using REDCap software, and exported to STATA version 15.0 (*StataCorp*, College Station, Texas, USA) for cleaning and subsequent analysis. To describe the prevalence of premalignant cervical lesions among HIV-positive and HIV-negative women attending the cervical cancer screening clinic at MRRH, the number of participants with premalignant lesions in each group was divided by the total number of participants in each group and expressed as a percentage. We used the chi-square test (χ2) to compare the prevalence of premalignant cervical lesions among HIV-positive and HIV-negative women. To establish the association between HIV status and premalignant cervical lesions, we used bivariate and multivariate logistic regression analyses to generate crude odds ratios (cORs) and adjusted odds ratios (aORs) with 95% confidence intervals (CIs), respectively. Variables with *p* < 0.2 in the bivariate analysis or those that were biologically plausible were entered into a multivariate logistic regression model to identify factors independently associated with premalignant lesions. *p* < 0.05 indicated statistically significant associations between the outcome and the independent variables at both the bivariate and multivariate analyses.

## Results

### Baseline social demographic characteristics

#### Study flow chart

**Fig. 2:**
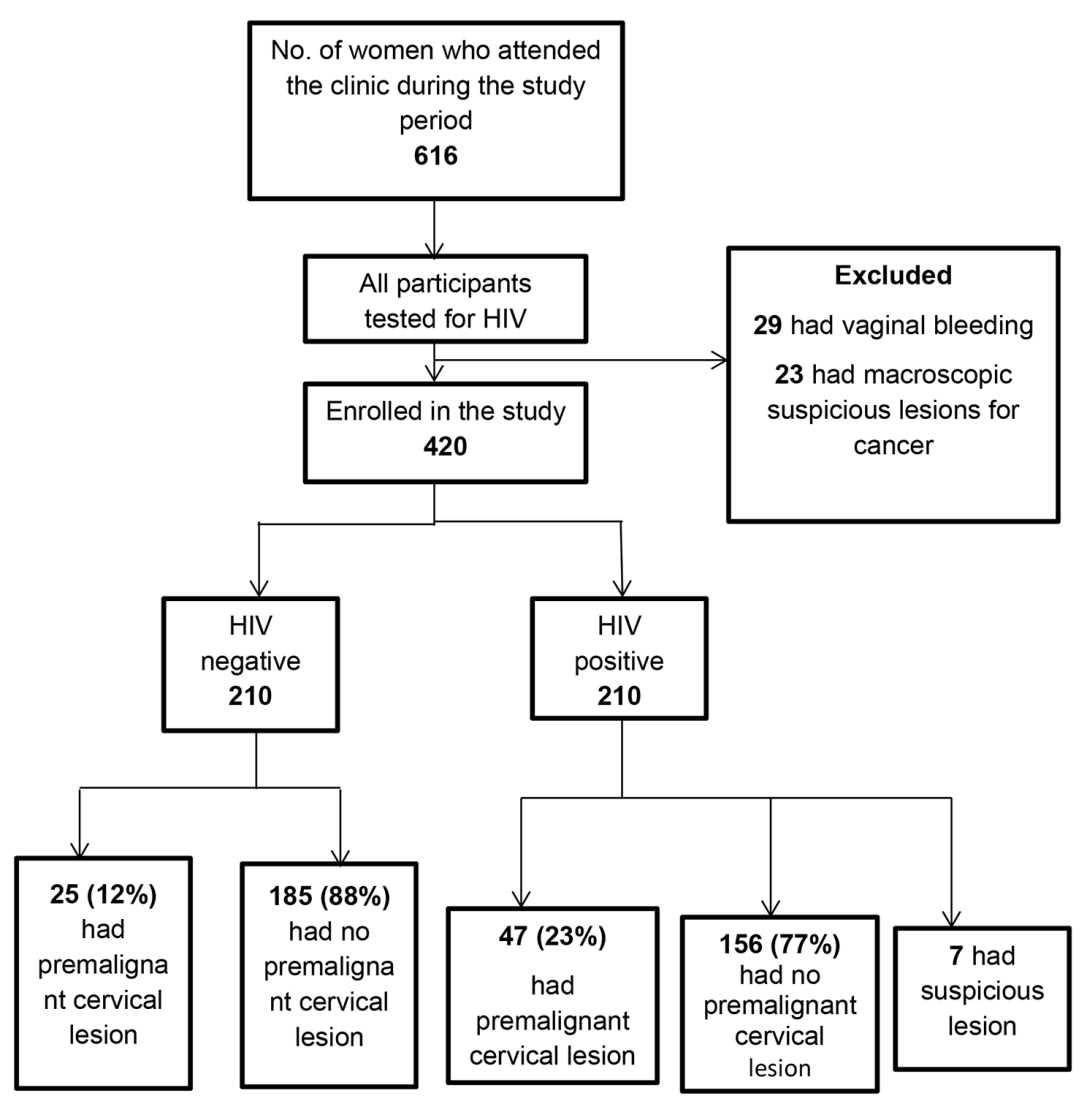
Study participants, Mbarara Regional Referral Hospital, Uganda, March–May, 2022

Among 616 women who attended the clinic during the study, 420 participants were enrolled using stratified sampling and excluded 29 women who had vaginal bleeding and 23 women who had suspicious lesions for cancer. Whereas the 7 HIV-positive women who had suspicious lesions for cancer on Pap smear were also excluded from the study (Fig. 2).

**Table 1:** Sociodemographic, medical and reproductive health characteristics of women attending cervical cancer screening at Mbarara Regional Referral Hospital, March–May 2022

Variable	Overall (*N* = 413)		HIV positive (*n* = 203)		HIV negative (*n* = 210)		*p*-value
	*n*	(%)	*n*	(%)	*n*	%	
Age* (Years)							0.002
< 30	46	(11)	15	(7)	31	(15)	
30–45	143	(35)	61	(30)	82	(39)	
> 45	224	(54)	127	(63)	97	(46)	
Marital status							< 0.001
Married	278	(67)	114	(56)	164	(78)	
Not married	135	(33)	89	(44)	46	(22)	
Religion							0.837
Christian	391	(95)	194	(96)	197	(94)	
Moslem	22	(5)	9	(4)	13	(6)	
Employment status							0.002
Farmer	228	(55)	124	(60)	104	(50)	
Business	99	(24)	52	(26)	47	(22)	
House wife	32	(8)	7	(3)	25	(12)	
Others	29	(7)	14	(8)	15	(7)	
Professional	25	(6)	6	(3)	19	(9)	
Level of Education							0.001
No formal education	67	(16)	38	(19)	29	(14)	
Primary	182	(44)	95	(47)	87	(41)	
Secondary	120	(29)	61	(30)	59	(28)	
Tertiary	44	(11)	9	(4)	35	(17)	
Residence							0.166
Rural	240	(58)	125	(62)	115	(55)	
Urban	173	(42)	78	(38)	95	(45)	
Ever Smoked							0.133
Yes	21	(5)	13	(6)	8	(4)	
No	392	(95)	190	(94)	202	(96)	
Body mass index (kg/m^2^)							0.204
Underweight < 18.5	22	(5)	8	(4)	14	(6)	
Normal (18.5–24.9)	203	(49)	109	(54)	94	(45)	
Overweight & obese ≥ 25	188	(46)	86	(42)	102	(49)	
History of STIs							< 0.001
Yes	203	(49)	80	(39)	123	(59)	
No	210	(51)	123	(61)	87	(41)	
Parity							0.265
0	12	(3)	3	(2)	9	(4)	
1–4	226	(55)	117	(58)	109	(52)	
≥ 5	175	(42)	83	(40)	92	(44)	
Abortion							0.056
Yes	156	(38)	86	(42)	70	(33)	
No	257	(62)	117	(58)	140	(67)	
Age at first sexual intercourse							< 0.001
< 18	173	(42)	111	(55)	62	(30)	
≥ 18	240	(58)	92	(45)	148	(70)	
No. of sexual partners in past 20 years							< 0.001
One	130	(32)	35	(17)	95	(45)	
≥two	283	(68)	168	(83)	115	(55)	
Oral contraceptive use							< 0.001
Yes	229	(55)	93	(46)	136	(65)	
No	184	(45)	110	(54)	74	(35)	

*Mean age (standard deviation) = (overall: 45.4 ± 11.6 years, HIV-positive: 46.9 ± 11.1 years, HIV-negative: 44.0 ± 11.9 years, STIs: sexually transmitted infections

The participants differed in age, marital status, employment status, and level of education. HIV-positive women were older than HIV-negative women, and women who were unmarried were more likely to be HIV positive. The distribution of employment types, marital status and education level differed significantly between the two groups. The mean age of all the participants was 45 years (± 11.6), as shown in Table 1. Most HIV-positive women had their first sexual intercourse at the age < 18 years, whereas most HIV-negative women started their first sexual intercourse at the age ≥ 18 years (54.8% vs. 70.5%, respectively). Compared with HIV-positive women, HIV-negative women had a greater history of STIs and had used combined oral contraceptives in the last 5 years. Compared with HIV-negative women, HIV-positive women had a history of two or more sexual partners in the last 20 years (Table 1).

#### Prevalence of premalignant lesions and associated factors

**Table 2:** Prevalence and distribution of premalignant cervical lesions by Pap smear results among women attending cervical cancer screening at Mbarara Regional Referral Hospital, March–May 2022

Pap smear result	Overall (*N* = 413)		HIV positive (*n* = 203)		HIV negative (*n* = 210)		*p*-value
	*N*	(%)	*n*	(%)	*n*	(%)	
Premalignant cervical lesions	72	(17)	47	(23)	25	(12)	0.003*
HSIL	8	(2)	7	(15)	1	(4)	0.001*
LSIL	55	(13)	35	(74)	20	(80)	0.001*
ASCUS	9	(2)	5	(11)	4	(16)	0.001*

ASCUS: atypical squamous cell of undetermined significance, LSIL: low-grade squamous intraepithelial lesion, HSIL: high-grade squamous intraepithelial lesion

The overall prevalence of premalignant cervical lesions in the study population was 17% (*n* = 72), with 23% (*n* = 47) in HIV-positive women and 12% (*n* = 25) in HIV-negative women (*p* < 0.003). The most common premalignant cervical lesions identified were low-grade squamous intraepithelial lesions (LSIL) in both HIV-positive (74%; *n* = 35) and HIV-negative women (80%; *n* = 20) (Table 2).

**Table 3:** Bivariate and multivariate analyses of factors associated with premalignant cervical lesions among women attending the cervical cancer screening clinic at Mbarara Regional Referral Hospital, March–May 2022

Characteristic	PML (*n* = 72)		No PML (*n* = 341)		cOR (95%CI)	*p*-value	aOR (95%CI)	*p*-value
	*n*	(%)	*n*	(%)				
Age (Years)								
< 30	9	(13)	38	(11)	Ref.		Ref.	
30–45	21	(29)	124	(36)	0.72 (0.3–1.69)	0.445	0.72 (0.27–1.95)	0.518
> 45	42	(58)	179	(53)	0.99 (0.45–2.21)	0.982	0.83 (0.3–2.32)	0.723
Marital status								
Not married	29	(40)	107	(31)	Ref.			
Married	43	(60)	234	(69)	0.68 (0.4–1.15)	0.146		
Religion								
Christian	65	(90)	323	(95)	Ref.			
Moslem	7	(10)	18	(5)	1.93 (0.76–4.81)	0.157		
Employment status								
Professional	4	(6)	22	(7)	Ref.			
House wife	4	(5)	29	(8)	0.76 (0.17–3.38)	0.717		
Farmer	39	(54)	184	(54)	1.17 (0.38–3.57)	0.788		
Business others	18 7	(25) (10)	83 23	(24) (7)	1.19 (0.37–3.89) 1.67 (0.43–6.53)	0.770 0.458		
Level of education								
No formal education	11	(15)	56	(16)	Ref.			
Primary	34	(47)	148	(44)	1.17 (0.56–2.47)	0.681		
Secondary	20	(28)	99	(29)	1.03 (0.46–2.3)	0.946		
Tertiary	7	(10)	38	(11)	0.94 (0.33–2.64)	0.903		
Residence								
Urban	30	(42)	146	(43)	Ref.			
Rural	42	(58)	195	(57)	1.05 (0.63–1.76)	0.858		
Ever Smoked								
No	66	(92)	324	(95)	Ref.		Ref.	
Yes	6	(8)	17	(5)	1.73 (0.66–4.56)	0.266	1.62 (0.53–4.92)	0.394
HIV status								
Negative	25	(35)	185	(54)	Ref.		Ref	
Positive	47	(65)	156	(46)	2.23 (1.31–3.79)	0.003*	2.37 (1.27–4.42)	0.007
Body mass index (BMI)								
Normal (18.5–24.9)	39	(55)	161	(47)	Ref.		Ref.	
Underweight (< 18.5)	7	(10)	15	(5)	1.93 (0.74–5.05)	0.182	2.3 (0.81–6.51)	0.117
Overweight/Obesity (≥ 25)	25	(35)	165	(48)	0.63 (0.36–1.08)	0.093	0.61 (0.34–1.1)	0.098
History of any other STIs								
No	42	(58)	170	(50)	Ref.			
Yes	30	(42)	171	(50)	0.71 (0.43–1.19)	0.192		
Parity								
0	4	(6)	9	(3)	Ref.		Ref.	
1–4	31	(43)	195	(57)	0.36 (0.1–1.23)	0.103	0.33 (0.08–1.37)	0.128
≥ 5	37	(51)	137	(40)	0.61 (0.18–2.08)	0.428	0.72 (0.17–3.15)	0.667
Abortion history								
No	46	(64)	211	(62)	Ref.			
Yes	26	(36)	130	(38)	0.92 (0.54–1.56)	0.749		
Age at first sexual intercourse								
≥ 18	34	(47)	205	(60)	Ref.		Ref.	
< 18	38	(53)	136	(40)	1.69 (1.01–2.81)	0.045	1.34 (0.75–2.4)	0.327
No. of sexual partners								
One	19	(26)	113	(33)	Ref.		Ref.	
≥two	53	(74)	228	(67)	1.38 (0.78–2.45)	0.266	1.08 (0.56–2.08)	0.822
Oral contraceptive use								
No	30	(42)	152	(45)	Ref.			
Yes	42	(58)	189	(55)	1.13 (0.67–1.89)	0.652		

PML = Premalignant cervical lesions, cOR = crude odds ratio, aOR = adjusted odds ratio, CI = confidence interval

According to our multivariate analysis, none of the sociodemographic factors were associated with premalignant lesions. HIV status was independently associated with premalignant cervical lesions in women attending the cervical cancer screening clinic at MRRH. An HIV-positive woman was approximately 2.4 times more likely to have premalignant cervical lesions (aOR: 2.37, 95% CI: 1.27–4.42; *p* = 0.007) (Table 3).

## Discussion

In this cross-sectional comparative study, approximately one-fifth (17%) of the women attending the cervical cancer screening clinic at MRRH had premalignant cervical lesions. The prevalence of premalignant cervical lesions was significantly higher among HIV-positive women than among HIV-negative women. Notably, a history of HIV infection was independently associated with increased odds of having premalignant cervical cancer lesions.

This finding is consistent with findings at Kampala International University Teaching Hospital in Uganda, which reported a similar prevalence of 21.9% [8]. Our findings are similar because the studies were both performed in teaching hospitals and they both used similar methods.

The prevalence of premalignant cervical lesions in the current study was higher in HIV-positive women than in HIV-negative women (23% versus 12%). This finding is consistent with findings at the Debre Markos Referral Hospital in Ethiopia, which found a prevalence of 17.8% in HIV-positive women and 10.3% in HIV-negative women [36]. Similarly, three hospitals in three regions of Swaziland also found a prevalence of 22.9% in HIV-positive women and 5.7% in HIV-negative women [17]. These studies were performed in low-income settings, such as our study, and low-income settings are characterized by gaps in the utilization and availability of healthcare, such as the low uptake of cervical cancer screening services [37]. Additionally, precancerous cervical lesions are aggressive and progress rapidly in immune-compromised patients because HIV-positive women are more likely to be infected with HPV and to have persistent HPV infection leading to precancerous lesions compared with HIV-negative women [19]. However, a study at the maternal and child healthcare hospital in Brazil found a lower prevalence in the HIV-positive group and HIV-negative group (12.1% versus 5.4% respectively) [38], and a study at the University of Nigeria Teaching Hospital (UNTH) in Enugu, Southeastern Nigeria, reported a prevalence of infection (12.6%) versus 4.6% (𝑃<0.014) [33]. This could have been because all the HIV-infected participants were receiving highly active antiretroviral therapy (HAART), which increases the CD4 count and hence reduces HPV persistence, leading to a low risk of premalignant cervical lesions. However, a tertiary hospital in North Central Nigeria found a higher prevalence of 56% in HIV-positive women and 13% in HIV-negative women [39], and another retrospective study in Tanzania showed a higher prevalence of 71.8% in HIV-positive women and 27.3% in HIV-negative women [40]. This could be due to differences in the methods used and the duration of the study.

All categories of premalignant cervical lesions identified in this study occurred more frequently among HIV-positive women, and the most common category was LSIL in both groups. This finding is similar to that reported at Maternal and Child Healthcare Hospital in Brazil, which found 6.4% LSIL in HIV-positive women and 2.7% LSIL in HIV-negative women; only one case of HSIL was found in all smears analyzed, and this patient was HIV positive [38]. Precancerous cervical lesions are considered to be more aggressive and progress rapidly in immune-compromised patients. Compared with HIV-negative women, HIV-positive women are more likely to be infected with HPV and to have persistent HPV, leading to larger and more difficult-to-treat precancerous lesions [17]. In HIV-negative women with competent immune systems, most infections are cleared spontaneously because of a cell-mediated immune response regulated by CD4 + lymphocytes [16].

A history of HIV infection was independently associated with premalignant cervical lesions in this study. HIV-positive women were approximately 2.4 times more likely to have premalignant cervical lesions than HIV-negative women. Compared with HIV-negative women, HIV-positive women have an increased risk of HPV infection and precancerous lesions [19]. This finding is consistent with findings at three hospitals in three regions of Swaziland [17]. Similarly, in a retrospective study from Tanzania on the influence of HIV/AIDS infection on precancerous cervical lesions, HIV infection was shown to increase the risk of premalignant cervical lesions [40]. Suggested explanations include a decrease in T-cell surveillance in HIV infection, which allows HPV replication, allowing for persistence of HPV infection, accumulation of mutations in the infected cells, and subsequent proliferation of dysplastic cells [20]. HIV alters the natural history of HPV infection, with decreased regression rates and more rapid progression to high grade and invasive lesions, which are refractory to treatment, requiring interventions that are more stringent including monitoring [22]. HIV-associated premalignant cervical lesions are thought to progress through the microsatellite instability pathway, whereas HIV-negative lesions progress through loss of heterozygosity. Interactions are likely via viral proteins, with HIV proteins enhancing the effectiveness of HPV proteins and possibly contributing to cell cycle disruption.

### Study strengths

This study had several strengths. We used cytology convention method where we captured both endocervical and ectocervical cells; these cells were subjected to quality control by another independent pathologist from another hospital. In addition, participants were screened for their HIV status and also the comparativeness of the design.

### Study limitations

We were able to answer our study objectives however; the study dataset had some missing viral load and HAART data. Additionally, we did not test for the presence of HPV or its effect on premalignant cervical lesions.

## Conclusions

We found a high prevalence of premalignant cervical lesions in women attending the MRRH cervical cancer screening clinic. Low-grade squamous intraepithelial lesions were the most common premalignant cervical lesions among all the women who participated in this study. The presence of all forms of premalignant cervical cancer lesions in women was positively associated with being HIV-positive. These findings further emphasize the need for the integration of HIV care and cervical cancer prevention programs. We recommend a longitudinal study to determine the outcomes of premalignant cervical lesions among HIV-positive women.

## Data Availability

For confidentiality reasons, the datasets used are not publicly available. However, the datasets used and/or analyzed during the current study are available from the corresponding author upon reasonable request (Dr. Justus Kirabira, Email: kjustus.justus19@gmail.com).

## Abbreviations

AIDS: Acquired Immune-Deficiency Syndrome
ASC: Atypical Squamous Cell
ASC-H: Atypical Squamous Cell that cannot exclude High-grade Lesion
ASC-US: Atypical Squamous Cell of Undetermined Significance
BMI: Body Mass Index
CD4: cluster of differentiation 4
CIN: Cervical Intraepithelial Neoplasia
DNA: deoxyribonucleic acid
HAART: highly active antiretroviral therapy
HIV: Human Immune-Deficiency Virus
HPV: human papilloma virus
HSIL: High Grade Squamous Intraepithelial Lesion
LSIL: Low Grade Squamous Intraepithelial Lesion
MRNA: messenger ribonucleic acid
MRRH: Mbarara Regional Referral Hospital
MUST: Mbarara University of Science and Technology
Pap Smear: Papanicolaou Smear
SD: standard deviation
STIs: Sexually Transmitted Infections
WHO: World Health Organization
Cor: Crude Odds Ratio
aOR: Adjusted Odds Ratio
